# Functional chimeras of flagellar stator proteins between *E. coli* MotB and *Vibrio* PomB at the periplasmic region in *Vibrio* or *E. coli*

**DOI:** 10.1002/mbo3.240

**Published:** 2015-01-29

**Authors:** Yuuki Nishino, Yasuhiro Onoue, Seiji Kojima, Michio Homma

**Affiliations:** Division of Biological Science, Graduate School of Science, Nagoya UniversityChikusa-ku, Nagoya, 464-8602, Japan

**Keywords:** Basal body, flagellar motor, T ring, *V. alginolyticus*

## Abstract

The bacterial flagellar motor has a stator and a rotor. The stator is composed of two membrane proteins, MotA and MotB in *Escherichia coli* and PomA and PomB in *Vibrio alginolyticus*. The *Vibrio* motor has a unique structure, the T ring, which is composed of MotX and MotY. Based on the structural information of PomB and MotB, we constructed three chimeric proteins between PomB and MotB, named PotB_91_, PotB_129,_ and PotB_138_, with various chimeric junctions. When those chimeric proteins were produced with PomA in a Δ*motAB* strain of *E. coli* or in Δ*pomAB* and Δ*pomAB* Δ*motX* strains of *Vibrio*, all chimeras were functional in *E. coli* or *Vibrio*, either with or without the T ring, although the motilities were very weak in *E. coli*. Furthermore, we could isolate some suppressors in *E. coli* and identified the mutation sites on PomA or the chimeric B subunit. The weak function of chimeric PotBs in *E. coli* is derived mainly from the defect in the rotational switching of the flagellar motor. In addition, comparing the motilities of chimera strains in Δ*pomAB*, PotB_138_ had the highest motility. The difference between the origin of the *α*1 and *α*2 helices, *E. coli* MotB or *Vibro* PomB, seems to be important for motility in *E. coli* and especially in *Vibrio*.

## Introduction

Many bacteria can swim using flagella. Each flagellum consists of a filament, a hook, and a basal body. The rotation of a flagellum is made by the interaction between the rotor and the stator (Morimoto and Minamino 2014). The rotor is called the C ring, which is associated beneath the basal body, and is composed of FliG, FliM, and FliN. Multiple stator units assemble around each rotor. There are two types of flagellar motors depending on the coupling ion. One is a proton driven motor with a stator composed of MotA and MotB, and is found in bacteria such as *Escherichia coli* or *Salmonella*. The other is a Na^+^ driven motor with a stator composed of PomA and PomB, and is found in such bacteria as *Vibrio alginolyticus* (Yorimitsu and Homma 2001; Blair 2003; Li et al. 2011b). MotA and PomA are four transmembrane (TM) domain proteins and MotB and PomB are one TM domain proteins (Chun and Parkinson 1988; Asai et al. 1997). PomAB or MotAB form heterohexameric channel complexes with an A4:B2 stoichiometry to conduct sodium ions or protons (Sato and Homma 2000; Kojima and Blair 2004; Takekawa et al. 2013). The stator A subunit has a cytoplasmic loop between TM3 and TM4 and charged residues in this loop interact with the C ring component FliG (Zhou et al. 1998b; Morimoto et al. 2010, 2013; Takekawa et al. 2014). The negative charged residue, D24 of PomB or D32 of MotB, is critical for the force generation and constitutes the ion-binding site in the stator channel (Zhou et al. 1998b; Sudo et al. 2009; Terashima et al. 2010b). It has been reported that a specific region, residues 44–58 in *Vibrio* PomB and 52–65 in *E. coli* MotB, serves as a plug, which regulates ion influx (Hosking et al. 2006; Kojima et al. 2009; Li et al. 2011a). Torque is generated by the interaction between the stator component PomA (MotA) and the rotor component FliG (Zhou et al. 1998a; Yakushi et al. 2006).

In *E. coli* and in *Salmonella*, the basal body is composed of the rod and several ring structures, the L, P, MS, and C rings (Macnab 1992). In *V. alginolyticus*, the basal body has a T ring and an H ring in addition to the rings found in *E. coli* (Terashima et al. 2006, 2010a). The T ring is composed of MotX and MotY. When MotX or MotY is deleted, the stator cannot assemble around the rotor or the basal body, indicating that the T ring is required for stator incorporation into the motor (Terashima et al. 2006). MotX affects the membrane localization of PomB, suggesting that PomB interacts with MotX, however, their direct binding has not yet been detected (Okabe et al. 2005). Some crystal structures of fragments of the stator B subunits from *Helicobacter pylori* and *Salmonella* have been resolved (Roujeinikova 2008; Kojima et al. 2009). The crystal structure of the periplasmic region of PomB (PomB_C_) has been recently resolved (Zhu et al. 2014). The N-terminus of PomB_C_ contains six negatively charged residues on the *α*1 and *α*2 helices and it forms a negatively charged surface. Interestingly, such a negative surface is not observed in the MotB_C_ structure of *Salmonella* (Kojima et al. 2009). The isoelectric point (pI) of MotX has been estimated at 8.48, raising the possibility that these negatively charged residues of PomB_C_ are involved in the interaction between PomB and MotX. Note that the estimated pI values of MotX proteins vary among bacteria, suggesting that MotX does not always have a positive charge. The charged residues might cause electrostatic interactions between these helices of PomB and MotX. To test this idea, we made charge reversal point mutants in the *α*1 helix or *α*2 helix of PomB_C_. The single mutations or the quintuple mutation conferred a swimming ability similar to the wild-type protein when induced by 0.02% arabinose, suggesting that those charge residues of the *α*1 or *α*2 helices are not critical for motor function (Zhu et al. 2014). In this study, we made several new chimeric proteins whose junction sites were designed at the N terminus of the *α*1 helix to the C terminus of the *α*2 helix. We used those chimeric proteins to determine the specificity of the B subunit of the flagellar motor protein for *Vibrio* and *E. coli*.

## Experimental Procedures

### Bacterial strains, plasmids and growth conditions

The bacterial strains and plasmids used in this study are shown in Table1. *Vibrio alginolyticus* was cultured in VC broth [0.5% (w/v) Polypeptone, 0.5% (w/v) Bacto yeast extract, 0.4% (w/v) K_2_HPO_4_, 3% (w/v) NaCl, 0.2% (w/v) d-glucose] or in VPG medium [1% (w/v) Polypeptone, 0.4% K_2_HPO_4_, 3% (w/v) NaCl, 0.5% glycerol] at 30°C. *Escherichia coli* was cultured in LB broth [1% (w/v) Bactotryptone, 0.5% (w/v) Bacto yeast extract, 0.5% (w/v) NaCl] at 37°C or TG broth [1% (w/v) Bactotryptone, 0.5% (w/v) NaCl, 0.5% (w/v) glycerol] at 30°C. Chloramphenicol was added to a final concentration of 2.5 *μ*g/mL for *V. alginolyticus* and 25 *μ*g/mL for *E. coli*.

**Table 1:** Strains and plasmids used in this study

Strain or plasmid	Description	Source or reference
*Escherichia coli*		
DH5*α*	Recipient for cloning experiments	
RP6894	Δ*motAB*	J. S. Parkinson
*Vibrio alginolyticus*		
NMB191	Δ*pomAB*	Yorimitsu et al. (1999)
TH4	Δ*pomAB* Δ*motX*	Terashima et al. (2006)
Plasmid		
pBAD33	Cm^r^, P*BAD*	Guzman et al. (1995)
pHFAB	pBAD33/*pomA*, *pomB*	Fukuoka et al. (2005)
pTF9	pBAD33/*pomA*, *potB59*	H. Fukuoka
pTSK62	pBAD33/*pomA*, *potB91*	This study
pTSK63	pBAD33/*pomA*, *potB129*	This study
pTSK64	pBAD33/*pomA*, *potB138*	This study

Cm^r^, chloramphenicol resistant; P*BAD*; arabinose promoter.

### Swimming assay in soft agar plates

Cells were inoculated in VPG soft agar plates [1% (w/v) Polypeptone, 0.4% K_2_HPO_4_, 3% (w/v) NaCl, 0.5% glycerol, 0.25% or 0.3% (w/v) Bacto agar] containing 0.02% (w/v) arabinose or 0.2% (w/v) arabinose and 2.5 *μ*g/mL chloramphenicol for *V. alginolyticus* or in TB soft agar plates [1% (w/v) Bactotryptone, 0.5% (w/v) NaCl, 0.3% (w/v) Bacto agar] containing 0.02% (w/v) arabinose and 25 *μ*g/mL chloramphenicol for *E. coli*. The plates were incubated at 30°C for the desired times.

### Measurement of swimming speed

Overnight cultures were inoculated into VPG medium for *V. alginolyticus* and into TG broth for *E. coli* containing 0.2% (w/v) arabinose or 0.02% (w/v) arabinose at a 100-fold dilution. Cells were grown at 30°C for 4 h for *V. alginolyticus* and 6 h for *E. coli*. Swimming of the cells was observed using dark-field microscopy at a 15-fold dilution with the growth medium. Swimming cells were recorded and then analyzed using software for motion analysis (Move-tr/2D; Library Co., Japan) as described previously (Terauchi et al. 2011). Swimming speeds were determined from data of at least 20 individual cells except for PotB_138_-sp1 (*n* = 5).

### Measurement of switching fraction

The switching fraction was measured by the tethered cell assay. Overnight *E. coli* cultures were inoculated into TG broth containing 0.02% (w/v) arabinose at a 100-fold dilution. Cells were grown at 30°C for 4 h and washed with 10 mmol/L potassium-phosphate, 10 mmol/L lactic acid, 100 mmol/L NaCl, and 0.1 mmol/L EDTA, pH 7.0. Their flagella were sheared by passing multiple times through a needle syringe. Cells were then attached on glass slides pretreated with an anti-*E. coli* flagellin antibody (Nishiyama and Kojima 2012). Rotation of the cells was observed using phase-contrast microscopy and was recorded on a computer using Power director (Cyber Link, Japan). Rotational motion of the cells was analyzed using software for motion analysis (Move-tr/2D; Library Co.). The number of switching times was counted in rotating cells for 30 sec. The switching fraction was calculated as the proportion of the number of cells whose flagella showed switching more than once to that of all rotating cells. The switching fraction was determined from about 20 individual cells.

### Immunoblotting

Cells were resuspended in sodium dodecyl sulphate (SDS) loading buffer and boiled at 95°C for 5 min, and then samples were subjected to SDS-polyacrylamide gel electrophoresis (SDS-PAGE) and transferred to polyvinyliden fluoride (PVDF) membranes. Immunoblotting was performed as previously described using an anti-*Salmonella* MotB antibody (Kojima et al. 2008) or an anti-PomA antibody (Yorimitsu et al. 1999). Protein detection was carried out by chemiluminescence using an LAS-3000 (Fujifilm Co., Japan).

### Suppressor isolation

*Escherichia coli* RP6894 (Δ*motAB*) carrying plasmids encoding PotB_91_ (pTSK62), PotB_129_ (pTSK63) or PotB_138_ (pTSK64) were freshly inoculated on TB soft agar plates containing 25 *μ*g/mL and 0.02% arabinose. Incubation at 30°C was continued overnight, and the motility halos that emerged from each inoculation were isolated and formed single colonies. After confirmation of the enhanced motility of the isolated suppressors, the plasmids were purified from these strains and were then sequenced to identify the mutation sites. DNA sequencing was performed with an ABI Prism genetic analyzer (Applied Biosystems, Japan).

## Results

### Motility of *Vibrio* by the chimeric proteins

**Figure 1:**
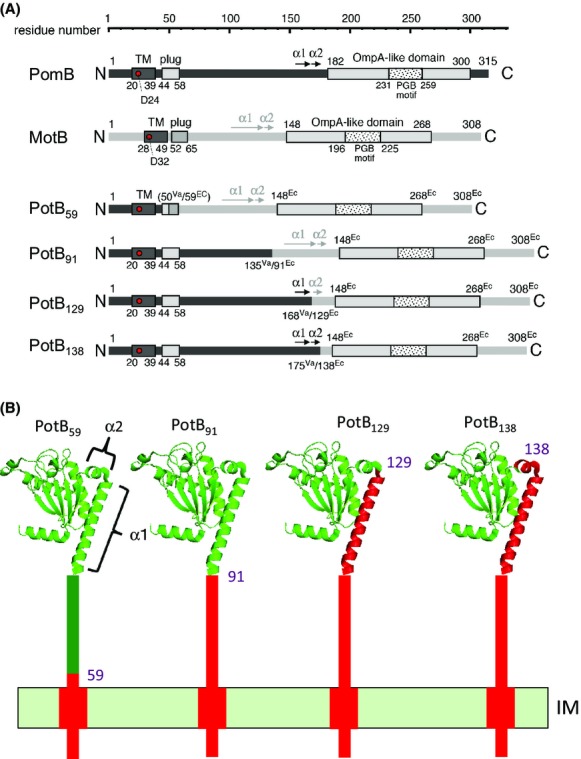
Constructs of the chimeric proteins used in this study. (A) Schematics of the primary structures of PomB, PotB_59_, PotB_91_, PotB_129_ and PotB_138_. PomB is composed of 315 amino acids and has a single TM domain (residues 20–39) in the N-terminal region, a plug domain (residues 44–58) and a large periplasmic region including an “Omp-A like domain” (residues 182–300). Asp-24, which is essential for ion translocation across the cytoplasmic membrane is shown as a red circle. (B) Topology models of the chimeric proteins. PotB_91_, PotB_129_ and PotB_138_ contain the region prior to the *α*1 helix of PomB, the region from the N terminal to the *α*1 helix of PomB, and the region from the N terminal to the *α*2 helix of PomB, respectively, and the rest of the region is derived from MotB. In the topological models, red indicates regions derived from PomB and green indicates regions derived from MotB.

**Figure 2:**
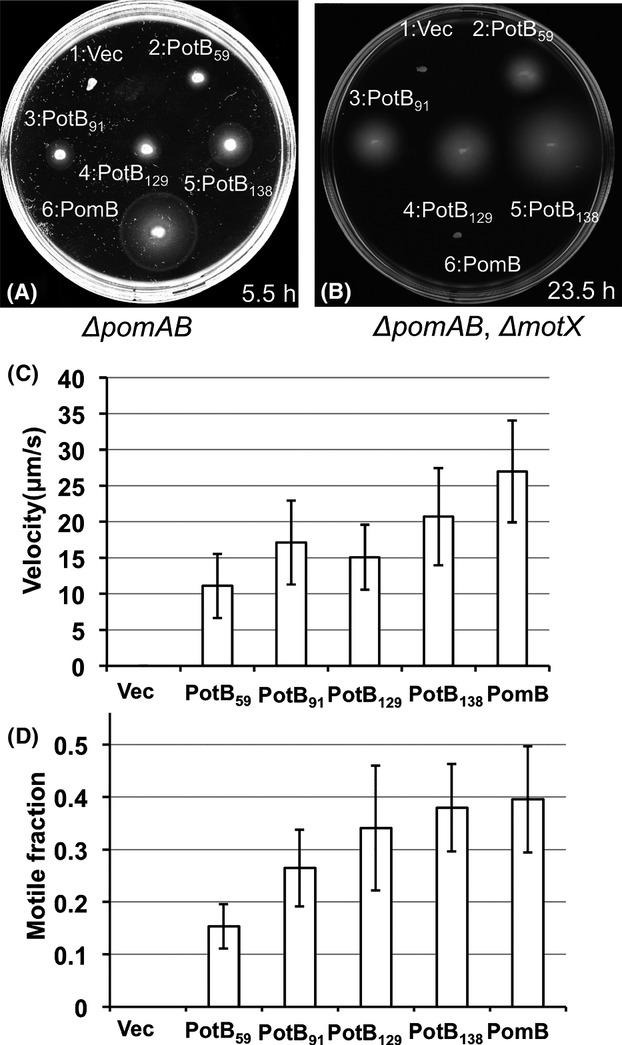
Motilities of chimeras in *Vibrio*. Fresh colonies of transformants from NMB191 (*Vibrio alginolyticus* Δ*pomAB*) (A) or TH4 (*V. alginolyticus* Δ*pomAB* Δ*motX*) (B) were inoculated and incubated on 0.25% VPG agar for 5.5 h (A) and on 0.3% VPG agar for 23.5 h (B) at 30°C with 0.02% arabinose. The cells have plasmids: 1, pBAD33 (Vec); 2, pTF9 (PotB_59_); 3, pTSK62 (PotB_91_); 4, pTSK63 (PotB_129_); 5, pTSK64 (PotB_138_) and 6, pHFAB (PomB) and produce wild-type PomA and the PotB chimera or the PomB proteins. Swimming speeds (C) or motile fractions (D) of cells producing the wild-type PomA and PotB chimera or PomB proteins in NMB191 (*V. alginolyticus* Δ*pomAB*) were measured. After 4 h culture in VPG at 30°C with 0.02% (w/v) arabinose, the cells were observed under a microscope.

We examined the roles of the *α*1 helix and the *α*2 helix using chimeric proteins composed of the N-terminus of PomB fused with the C-terminus of MotB. We previously showed that a chimeric protein, PotB, renamed as PotB_59_, that has the N-terminal region of PomB (1–50) fused to the C-terminal periplasmic region of *E. coli* MotB (59–308), is functional with PomA in *E. coli* as well as in strains of *V. alginolyticus* that are defective in *motX* or *motY* (Asai et al. 2003). Based on the structure comparison between PomA and MotB to determine the junction sites (Kojima et al. 2009; Zhu et al. 2014), we made three new chimeric proteins, PotB_91_, PotB_129_ and PotB_138_ (Figs.1, S1). PotB_91_ consists of PomB (1–135) and MotB (91–308), PotB_129_, consists of PomB (1–168) and MotB (129–308), and PotB_138_ consists of PomB (1–175) and MotB (138–308) (Fig.1). We introduced these proteins with PomA into the mutant strains of *V. alginolyticus* Δ*pomAB* (NMB191) or Δ*pomAB* Δ*motX* (TH4) and observed their motilities in soft agar plates (Fig.2A and B). All three chimeric proteins conferred motile ability to *V. alginolyticus* even in the absence of the T ring, however, the original stator complex composed of PomA and PomB did not confer any motility in the absence of MotX or the T ring as previously reported (Asai et al. 2003). The sizes of the swimming rings without MotX or the T ring by the chimeric stators were much smaller than in the T ring containing strain. The sizes of the swimming rings of PotB_91_, PotB_129_, and PotB_138_ were larger than that of PotB_59_ and the diameter of PotB_138_ was the largest among the three chimeric proteins. These observations suggest that the *α*1 helix and especially the *α*2 helix of PomB are important for function in *Vibrio*.

We next investigated the swimming ability in liquid medium (Fig.2C and D). PotB_91_, PotB_129_, and PotB_59_ in *V. alginolyticus* Δ*pomAB* conferred similar swimming speeds, about 15–20 *μ*m/sec, and this speed was about half of the wild-type PomB. The swimming speed of PotB_138_ was faster than the other chimeric proteins and these swimming speeds correlated with the diameters of the swimming rings. The chimeric proteins in the *V. alginolyticus* Δ*pomAB* Δ*motX* strain did not confer motility in most cells under the same conditions as Δ*pomAB*.

### Expression profiles of the chimeric proteins in *Vibrio*

We examined the expression and stability of the chimeric proteins by immunoblotting using antibodies against MotB and PomA (Fig. S2). In regard to PomB or the chimeras, all chimeric proteins were detected but a band was not detected in the vector control or in wild-type PomB from the pHFAB plasmid in Δ*pomAB* Δ*motX*. The PomB protein was not detected by the anti-MotB antibody. The band intensity of PotB_91_ was as high as that of PotB_138_, but the expression level of PotB_129_ was very low in both hosts. This may suggest that the PotB_129_ protein is unstable or is difficult to express. In contrast to the amounts of B subunits, the band intensities of PomA in the hybrid strains were similar in both hosts. The production of PomA was not affected by the chimeric *potB* genes.

### Motility of *E. coli* by the chimeric proteins

**Figure 3:**
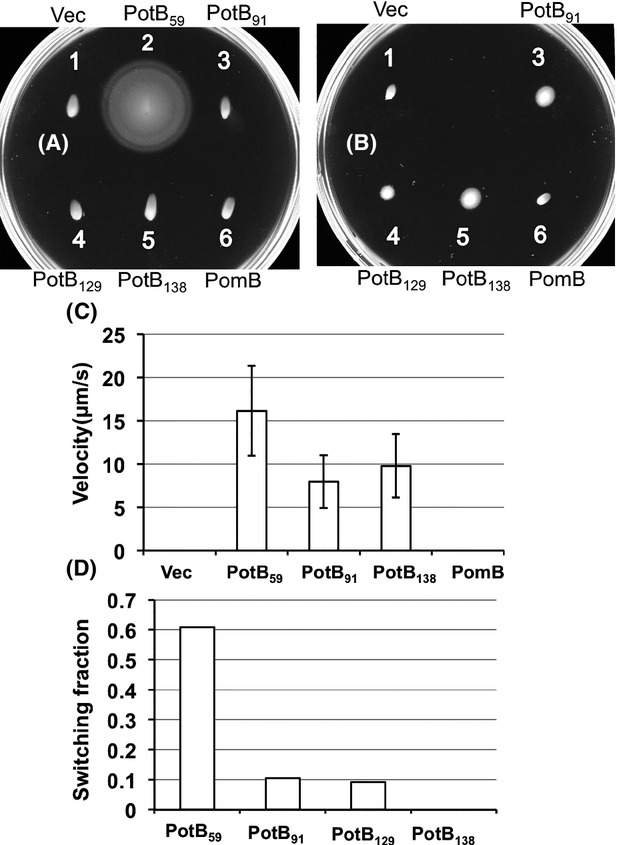
Motilities of chimeras in *Escherichia coli*. Fresh colonies of RP6894 (*E. coli* Δ*motAB*) were inoculated and incubated on 0.3% TB agar for 13 h (A) and 20 h (B) at 30°C. Each strain has the following plasmid: 1, vector plasmid pBAD33 (Vec); 2, pTF9 (PotB_59_); 3, pTSK62 (PotB_91_); 4, pTSK63 (PotB_129_); 5, pTSK64 (PotB_138_); 6, pHFAB (PomB). (C) Swimming speeds of the chimera strains of RP6894 were measured. After 6 h of culture in TG medium with 0.02% (w/v) arabinose, the cells were diluted 15-fold with TG medium. Swimming speeds were measured as described in Experimental Procedures. (D) Rotational switching of the flagellar motor observed by the tethered cell assay. “Switching fraction” means the proportion of the cell number of rotating cells who changed the direction at least one time in 30 sec against the total number of rotating cells.

We found that all strains produced chimeric proteins which conferred motility in the *motX* deficient strain, *V. alginolyticus* Δ*pomAB* Δ*motX*. Thus, we next examined the influences of the swimming abilities if the chimeric proteins were expressed in *E. coli* Δ*motAB*. The swimming abilities of chimera strains in soft agar plates were observed (Fig.3A and B). Only PotB_59_ showed swimming motility clearly in soft agar plates and the other chimera strains did not confer swimming rings even after 13 h of incubation in soft agar plates containing 0.25% agar. In regard to the swimming ability in liquid media, we examined the swimming speeds for all strains producing chimeric proteins (Fig.3C). PomB did not confer any motility even after overnight incubation in soft agar plates. Against our assumption, the swimming speeds of PotB_91_ and PotB_138_ were half as much as that of PotB_59_. This may suggest that the force-generating ability is not so different between PotB_91_ or PotB_138_ and PotB_59_. Next, because the swimming motilities of the new chimera strains on soft agar plates were different from that in liquid medium, we inferred that the reason for this difference might not be the swimming speed or torque. We thought that the most likely reason might be a defect in rotational switching, so that cells exhibited an imbalanced transition between smooth swimming – tumble states in the motility agar plates. When flagella, which are helical filaments, rotate counter-clockwise (CCW), cells show smooth swimming and when flagella rotate clockwise (CW), cells tumble. So, we examined the flagellar-rotational switching ability of the chimera strains using a tethered cell assay, in which we can detect rotational direction directly at the single motor level (Fig.3D). The switching ability of PotB_59_ was high since 60% of the cells could change directions in 30 sec. On the other hand, the switching abilities of PotB_91_ and PotB_129_ were less than 10% of PotB_59_ and that of PotB_138_ was not detectable in these conditions. All the chimeric motors rotated with a CCW bias. Therefore, we concluded that the difference between swimming on soft agar plates and in liquid medium of PotB_91_ and PotB_138_ strains was mainly caused by a deficiency of the switching property. We detected chimeric proteins in hybrid *E. coli* strains by immunoblotting (Fig. S3). The band intensities of the PotB_91_ and PotB_138_ bands were comparable to PotB_59_, but the intensity of the PotB_129_ band was much less than the others. Thus, the expression profiles of chimeric proteins in *E. coli* are similar to those in *Vibrio*.

### Suppressor mutants from the chimeras

**Figure 4:**
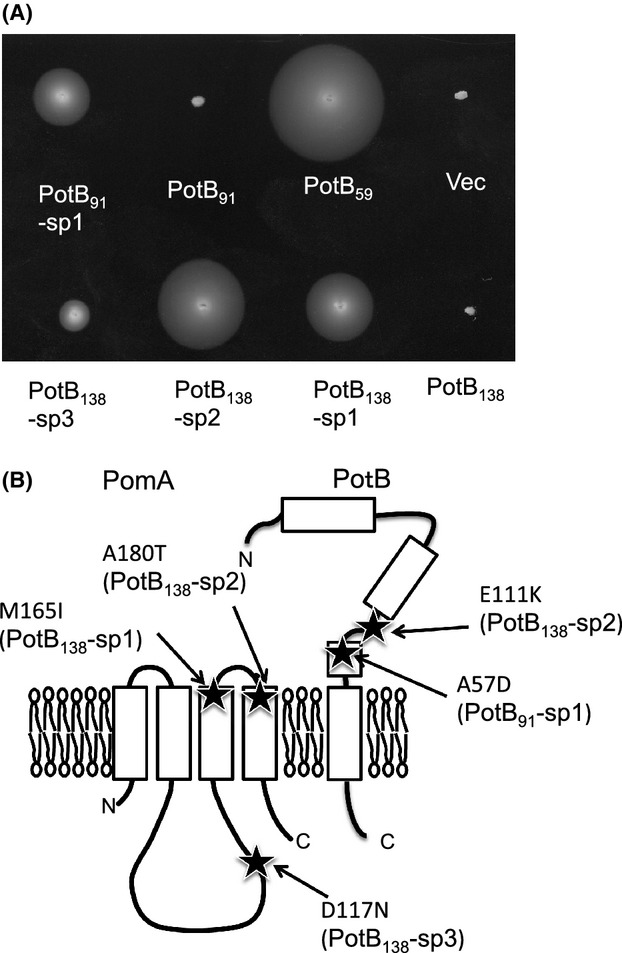
Suppressor mutants isolated from the chimera strains. (A) Swimming abilities in soft agar plates of chimeras in *Escherichia coli* were examined. Fresh colonies of RP6894 (*E. coli* Δ*motAB*), which were retransformed with the plasmids recovered from the suppressor strains, were inoculated and incubated on 0.3% TB agar for 22–24 h at 30°C with 0.02% arabinose. 1, vector plasmid pBAD33 (Vec); 2, pTF9 (PotB_59_); 3, pTSK62 (PotB_91_); 4, PotB_91_-sp1; 5, pTSK64 (PotB_138_); 6, PotB_138_-sp1; 7, PotB_138_-sp2; 8, PotB_138_-sp3. (B) The mutation sites of the suppressors mapped on a topology model of PomA and PomB.

Because of the results for *E. coli* Δ*motAB*, we isolated spontaneous motile suppressors from the hybrid *E. coli* strains that were almost nonmotile in soft agar plates (Fig.4A). We isolated suppressors that showed significantly improved motility on soft agar plates compared to the original strain, and most of the suppressor mutations were mapped on the plasmid encoding both *pomA* and *potB*. We sequenced the plasmids to determine the mutation site (Fig.4B). For PotB_138_, suppressor 1 (sp1) carries M165I in PomA, sp2 has two mutations, A180T in PomA and E111K in PotB, and sp3 has D117N in PomA. For PotB_91_, sp1 has A57D in PotB. The diameter of PotB_138_-sp2 was the largest among the three suppressors of PotB_138_ and the diameter of PotB_138_-sp3 was the smallest among them. All mutations in PotB chimeras were mapped on the PomB region, so that the residue number is the PomB numeration.

**Figure 5:**
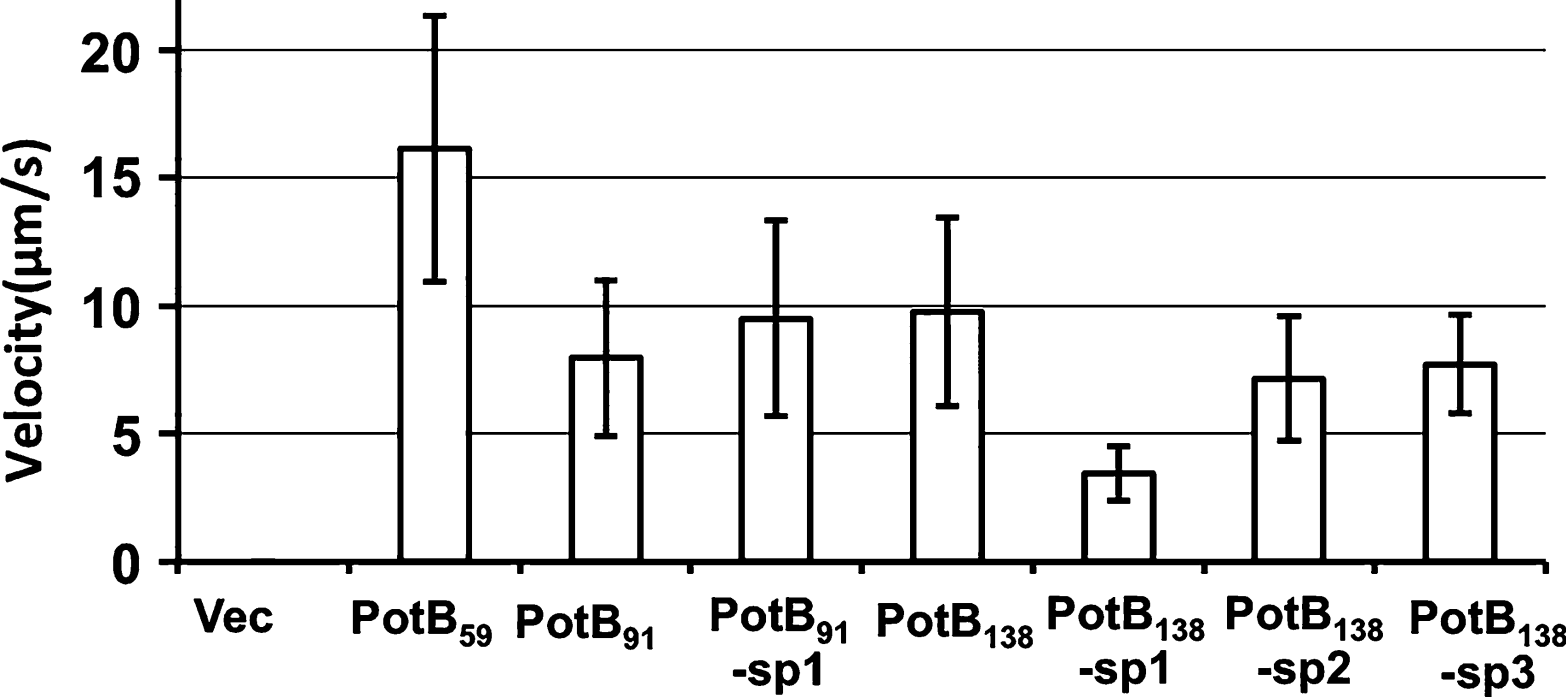
Swimming speeds of suppressors. After 4 h incubation in TG medium with 0.02% (w/v) arabinose, the cells were observed under a microscope. For details of this analysis, see Experimental Procedures.

We examined the swimming speeds in liquid media of these suppressors (Fig.5). The swimming speed of PotB_138_-sp1 was slower than PotB_138_, but the swimming speeds of PotB_138_-sp2 and PotB_138_-sp3 were as fast as PotB_138_. The swimming speed of PotB_91_-sp1 was a little faster than PotB_91_. The swimming rings in soft agar plates were much increased but the swimming speeds of the suppressors were not changed. Thus, the suppressor mutations of PotB_91_ and PotB_138_ are suggested to affect the rotational switching of the flagellar motor mainly but not the force generation by the stator and rotor interaction.

## Discussion

It was reported that the Δ*pomAB* mutant (*V. alginolyticus* NMB191), which carries a plasmid (pHFAB) containing the *pomA* and *pomB* genes, can swim well but that the Δ*pomAB* Δ*motX* mutant (*V. alginolyticus* TH4), which carries the plasmid pHFAB, cannot swim at all (Asai et al. 2003; Terashima et al. 2006). When MotX or MotY is missing, the stators composed of PomA and PomB cannot assemble around the flagellar rotor in *Vibrio*. A functional chimera PotB (renamed in this study as PotB_59_) with PomA conferred motility to either the Δ*pomAB* Δ*motX* mutant or the Δ*pomAB* mutant (Asai et al. 2003). The new chimeric proteins of PotB_91_, PotB_129_ and PotB_138_ conferred motility to either the Δ*pomAB* Δ*motX* mutant or the Δ*pomAB* mutant (Fig.2). The motility without MotX in soft agar plates is severely reduced and we could not detect swimming cells that were deficient MotX in liquid medium until 4 h after the overnight culture was inoculated into fresh broth at a 100-fold dilution. Without the T ring, the swimming fraction of PotB chimera strains is very small, and it is much smaller than that with the T ring. We assume that the PotB chimeric proteins of the stator can assemble around the rotor but the basal body without the T ring probably is not able to rotate smoothly. We cannot rule out that the PotB chimeric proteins are not efficiently assembled or installed into the motor in the T ring deficient cells.

Although the expression level of PotB_129_ was very low in the chimeric proteins (Fig. S2), PotB_129_ conferred swimming ability both in soft agar plates and in liquid media similar to PotB_91_. The swimming speed of PotB_138_ was comparable to that of the wild-type PomB. Although we cannot rule out that the difference in motility among chimera strains is mainly due to the low expression of PotB_129_, the expression of PotB_129_ seems to be sufficient to make adequate stators since the induction by arabinose 0.02% is high and the protein level is higher than the chromosomal expression level. Among the chimeric proteins, PotB_138_ conferred better motility than the others in the Δ*pomAB* mutant as well as in the Δ*pomAB* Δ*motX* mutant. The *α*1 and *α*2 helices of PotB_138_ are derived from *Vibrio* PotB_138_. This may suggest that the *α*1 and *α*2 helices are important for motility in *Vibrio* and we speculate that the helices or the region around the helices interact with MotX. We had speculated previously that the structure of an N-terminal region of PomB_C_ (the N terminal PEM region of PomB, 121–153) was disordered when the PomAB stator complex was activated (Zhu et al. 2014). The exact role of the helices remains unclear and it is possible that the helices interact with a flagellar protein other than MotX.

We have shown that PotB_59_ with PomA conferred motility on Δ*motAB E. coli* and that *E. coli* strains are driven by sodium ions and not by protons (Asai et al. 2003). The swimming motility of PotB chimera strains in *Vibrio* in soft agar plates was smaller than that of PomB. However, the swimming motility of PotB in liquid medium seems not to be different from that of PomB. The swimming speed in liquid medium is almost linearly proportional to the ratio of the swimming fraction except for PotB_129_. The protein expression level of PotB_129_ was very low compared to the other chimeras in both *E. coli* and *V. alginolyticus* (Fig. S2A). In contrast to *Vibrio*, *E. coli* with the new chimera PotB did not show swimming ability in soft agar plates but could swim in liquid medium. The tethered cell assay showed that their switching abilities were much worse than PotB_59_ (Fig.3D). The B subunit of the stator has a linker region from the region posterior to the plug to the region prior to the *α*1 helix. The linker regions of PotB_91_ or PotB_138_ are derived from PomB and the linker region of PotB_59_ is derived from MotB. The linker region may affect the switching between CCW and CW rotation which is believed to be caused by a conformational change of the FliG C-terminal region (Lloyd et al. 1999; Lee et al. 2010; Minamino et al. 2011). It is inferred that a specific interaction between the FliG C-terminal region and the cytoplasmic loop region of MotA or PomA determines the direction of the force generation (Zhou et al. 1998a; Yorimitsu et al. 2002; Paul et al. 2011; Takekawa et al. 2014). We have shown that some mutations of the charged residues of PomA and FliG cause a defect in the rotational switching of the motor (Takekawa et al. 2014). The suppressor mutations were isolated from PotB_91_ or PotB_138_ in *E. coli*. For PotB_138_, the mutations are M165I, D117N and A180T in PomA or E111K in PotB. For PotB_91_, the mutation is A57D in PotB. The mutations are located in the plug or the linker region of the PotB proteins, in the periplasmic boundary region of PomA TM3 or TM4, and in a cytoplasmic region of PomA. Because MotB_C_ is required not only for proper anchoring of the stator to its binding site on the motor (Blair et al. 1991) but also for proper alignment of the stator relative to the rotor (Garza et al. 1996), these genetic data suggest the possibility that the suppressor mutations may affect the stator–rotor interface of the PomA/PotB chimeric motor in *E. coli*, allowing the motor to spin in both the CCW and CW directions. We showed that MotB allows the *E. coli* MotA/B complex to efficiently function as a H^+^-coupled stator in *Vibrio* and hyper-motile suppressions were isolated in the periplasmic region of MotB (Asai et al. 2003). The mechanism was not assigned in that study, but the suppression may similarly occur as the suppression of this study.

## Supporting Information

Figure S1 Sequence alignment based on the secondary structures of chimeric proteins refer to the crystal structures of PomB_C5_ and MotB_C2_ (PDB ID codes: 3WPW and 2ZVY). They show junction sites of chimera strains and important motifs or domains. Blue underlines show *α* helices in regions whose crystal structures are solved and green underlines show *β* sheets. The black box shows the TM region and the purple box shows the plug region. **Figure S2.** Immunoblotting of chimeric proteins expressed in *Vibrio*. Whole cells of NMB191 (*Vibrio alginolyticus* Δ*pomAB*) and TH4 (*V. alginolyticus* Δ*pomAB* Δ*motX*) containing the vector plasmids pBAD33 (Vec), pTF9 (PotB_59_), pTSK62 (PotB_91_), pTSK63 (PotB_129_), pTSK64 (PotB_138_) or pHFAB (PomB) were subjected to SDS-PAGE, followed by immunoblotting using anti-*Salmonella* MotB_C_ (A) and anti-PomA antibodies (B). **Figure S3.** Immunoblotting of chimera proteins expressed in *Escherichia coli*. Whole cells of *E. coli* Δ*motAB* (RP6894) containing the vector plasmid pBAD33 (Vec), pTF9 (PotB_59_), pTSK62 (PotB_91_), pTSK63 (PotB_129_), pTSK64 (PotB_138_) or pHFAB (PomB) were subjected to SDS-PAGE and immunoblotting was performed using an anti-*Salmonella* MotB antibody.
