# Supplementary material for: Functional chimeras of flagellar stator proteins between E. coli MotB and Vibrio PomB at the periplasmic region in Vibrio or E. coli
Source: Microbiologyopen. 2015 Jan 29;4(2):323–31. doi: 10.1002/mbo3.240 (PMC4398512; doi:10.1002/mbo3.240)
Supplement: Figure S1 — Sequence alignment based on the secondary structures of chimeric proteins refer to the crystal structures of PomBC5 and MotBC2 (PDB ID codes: 3WPW and 2ZVY). They show junction sites of chimera strains and important motifs or domains. Blue underlines show α helices in regions whose crystal structures are solved and green underlines show β sheets. The black box shows the TM region and the purple box shows the plug region. Figure S2. Immunoblotting of chimeric proteins expressed in Vibrio. Whole cells of NMB191 (Vibrio alginolyticus ΔpomAB) and TH4 (V. alginolyticus ΔpomAB ΔmotX) containing the vector plasmids pBAD33 (Vec), pTF9 (PotB59), pTSK62 (PotB91), pTSK63 (PotB129), pTSK64 (PotB138) or pHFAB (PomB) were subjected to SDS-PAGE, followed by immunoblotting using anti-Salmonella MotBC (A) and anti-PomA antibodies (B). Figure S3. Immunoblotting of chimera proteins expressed in Escherichia coli. Whole cells of E. coli ΔmotAB (RP6894) containing the vector plasmid pBAD33 (Vec), pTF9 (PotB59), pTSK62 (PotB91), pTSK63 (PotB129), pTSK64 (PotB138) or pHFAB (PomB) were subjected to SDS-PAGE and immunoblotting was performed using an anti-Salmonella MotB antibody. [file mbo30004-0323-sd1.pdf]

```
              .........10 .........20 .........30 .........40 .........50
StMotB      1 MKNQAHPIVV VKRRRHKPHG GGAHGSWKIA YADFMTAMMA FFLVMWLISI  50
VaPomB      1 ---------M DDEDNKCDCP PPGLPLWMGT FADLMSLLMC FFVLLLSFSE  41
              .......10. .......20. .......30. .......40.

                      ↓59
              .........60 .........70        .. ........   80.........
StMotB     51 SSPKELIQIA EYFRTPLATA --------VT GGNRIAN--- SESPIPGGGD  89
VaPomB     42 MDVLKFKQIA GSMKFAFGVQ NQLEVKDIPK GTSIIAQEFR PGRPEPTPID  91
              .......50. .......60. .......70. .......80. .......90.
                    50↑

                   90↓91.......
StMotB     90 -------DYT QQQGEVE--- ---------- ---------- ----------  99
VaPomB     92 VIMQQTMDIT QQTLEFHEGE SDRAGGTKRD EGKLTGGQSP ATSTQNNESA 141
              ......100. ......110. ......120. ......130. ......140.
                                                     135↑

                                   α1                   ↓129α2
                         100....... 110....... 120....... 130.......
StMotB     88 ---------- --KQPNIDEL KKRMEQSRLN KLRGDLDQLI ESDPKLRALR 137
VaPomB    142 EADMQQQQSK EM-------- -----SQEME TLMESIKKAL E--REIEQ-- 174
              ......150. ..         .....160........ .170....
                                             α1 168↑     α2

              ↓138 β1      β2                              α3
              140....... 150....... 160....... 170....... 180.......
StMotB    138 PHLKIDLVQE GLRIQIIDSQ NRPMFKTGSA EVEPYMRDIL RAIAPVLNGI 187
VaPomB    175 GAIEVENLGQ QIVIRMR--- EKGAFPEGSA FLQPKFRPLV RQIAELVKDV 221
              ...180.... ...190.    ......200. ......210. ......220.
          175↑   β1        β2                              α3

                 β3                            α4
              190....... 200....... 210....... 220....... 230.......
StMotB    188 PNRISLAGHT DDFPYANGEK GYSNWELSAD RANASRRELV AGGLDNGKVL 237
VaPomB    222 PGIVRVSGHT DNRPLDS-EL YRSNWDLSSQ RAVSVAQEME KVRGFSHQRL 270
              ......230. .......240 .......250 .......260 .......270
                 β3                            α4

              β4                       β5         α5
              240....... 250  ..... ..260..... ..270..... ..280.....
StMotB    238 RVVGMAATMR LSD--RGPDD AINRRISLLV LNKQAEQAIL HENAESQNEP 285
VaPomB    271 RVRGMADTEP LLPNDSDDNR ALNRRVEISI MQGEPLYSEE VPVIQ----- 315
              .......280 .......290 .......300 .......310 .....
              β4                 αs    β5

              ..290..... ..300..... ....
StMotB    272 VSVLQQPAAA PPASVPTSPK AEPR 309
VaPomB        --------------------------
```

Fig. S1 Nishino et al.

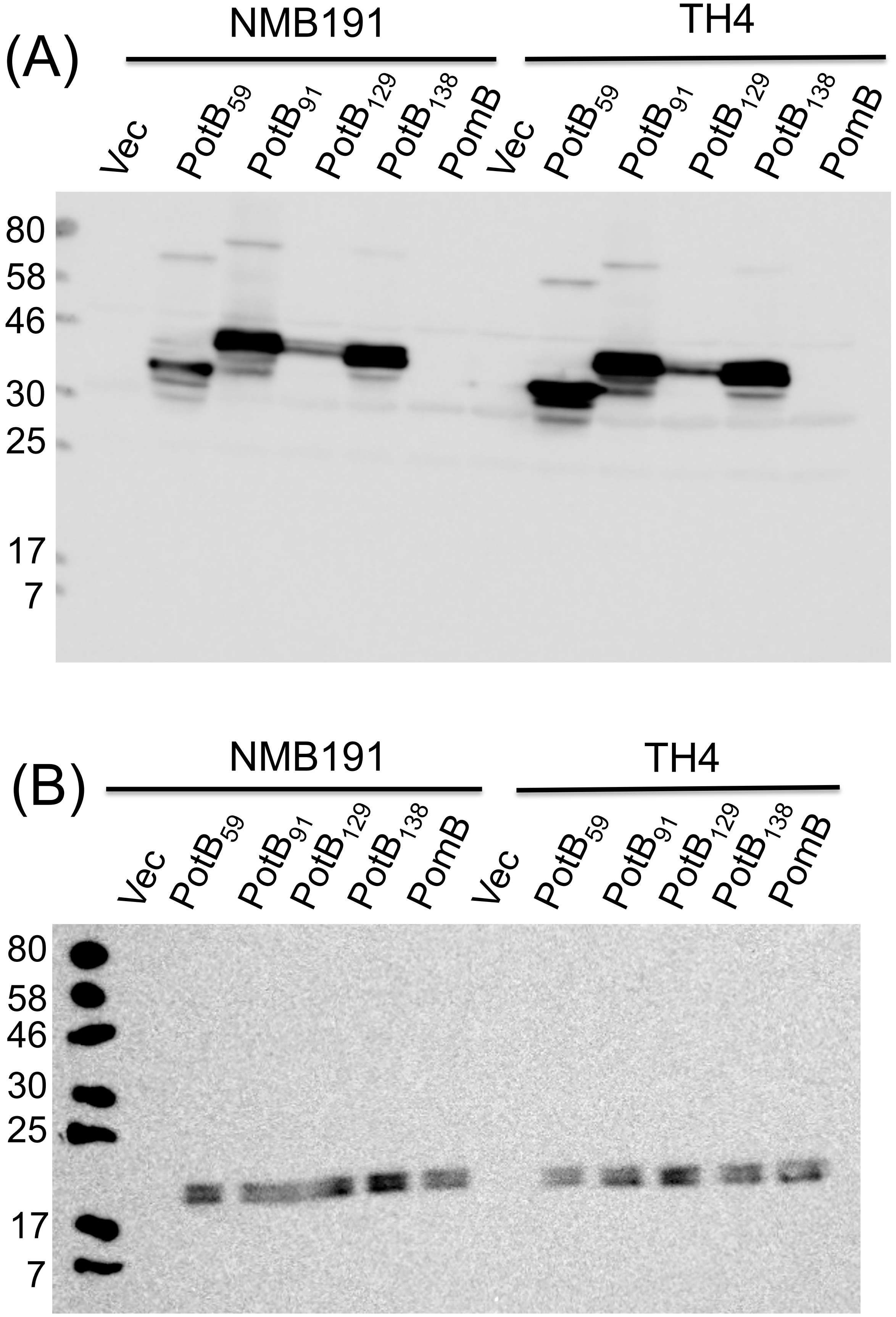

Fig. S2 Nishino et al.

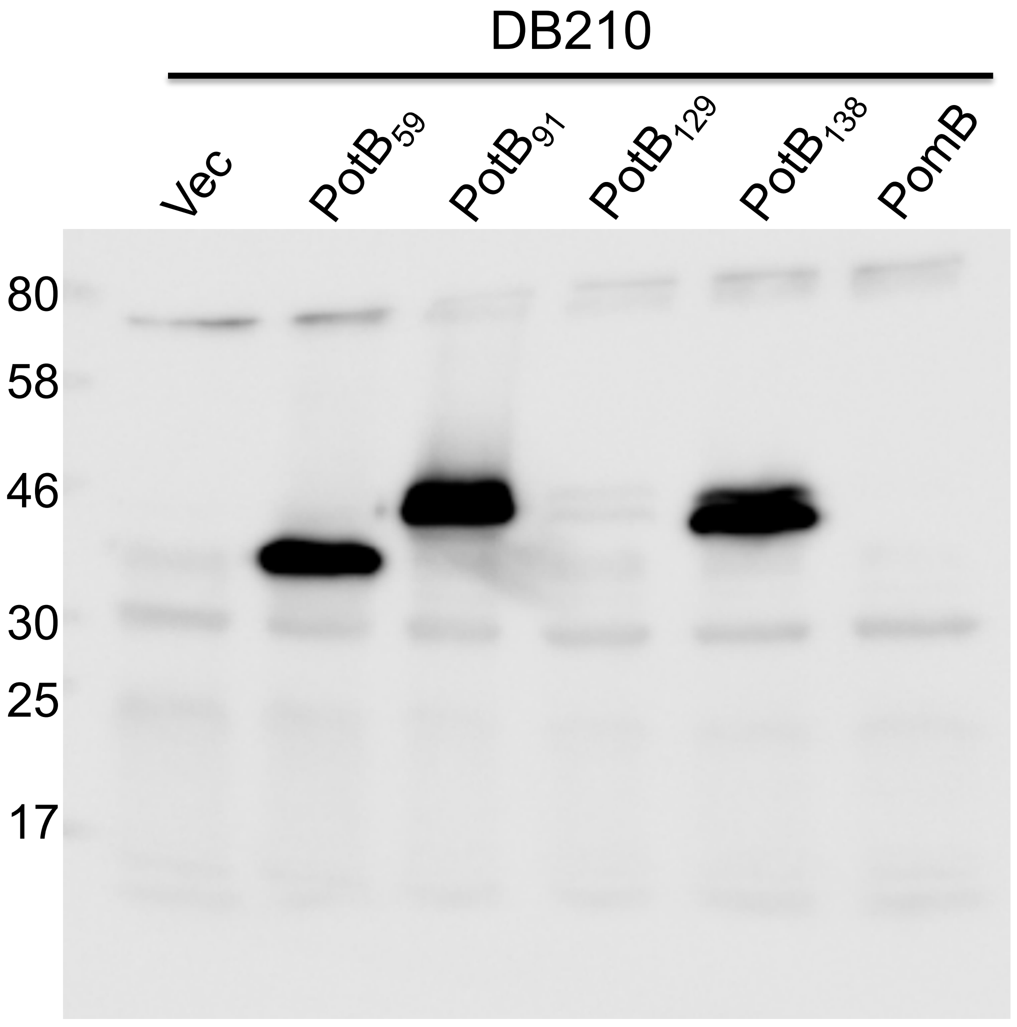

Fig. S3 Nishino et al.
